# Service utilization and cost of implementing a comprehensive HIV prevention and care program among people who inject drugs in Delhi, India

**DOI:** 10.1186/s12954-017-0165-y

**Published:** 2017-06-14

**Authors:** Mary Philip Sebastian, Aparajita Dasgupta, Lopamudra Ray Saraswati, Asha Singh, Vartika Sharma, Ira Madan, Waimar Tun, Julie Pulerwitz, Ibou Thior, Avina Sarna

**Affiliations:** 1 Ex-Population Council, Delhi, India; 2 Economics, Ashoka University, Sonepat, Haryana, India; 3 Population Council, India Habitat Center, New Delhi, 110003 India; 4 Sahara Center for Residential Care and Rehabilitation, New Delhi, India; 5 Population Council, Washington, DC USA; 6 PATH, Washington, DC USA

**Keywords:** HIV, PWID, Harm reduction, Needle-syringe program, HIV, Hepatitis B, Hepatitis C

## Abstract

**Background:**

WHO, UNODC, and UNAIDS recommend a comprehensive package for prevention, treatment, and care of HIV among people who inject drugs (PWID). We describe the uptake of services and the cost of implementing a comprehensive package for HIV prevention, treatment, and care services in Delhi, India.

**Methods:**

A cohort of 3774 PWID were enrolled for a prospective HIV incidence study and provided the comprehensive package: HIV and hepatitis testing and counseling, hepatitis B (HB) vaccination, syndromic management of sexually transmitted infections, clean needles-syringes, condoms, abscess care, and education. Supplementary services comprising tea and snacks, bathing facilities, and medical consultations were also provided. PWID were referred to government services for antiretroviral therapy (ART), TB care, opioid substitution therapy, and drug dependence treatment/rehabilitation.

**Results:**

The project spent USD 1,067,629.88 over 36 months of project implementation: 1.7% on capital costs, 3.9% on participant recruitment, 26.7% for project management, 49.9% on provision of services, and 17.8% on supplementary services. Provision of HIV prevention and care services cost the project USD 140.41/PWID/year. 95.3% PWID were tested for HIV. Of the HIV-positive clients, only 17.8% registered for ART services after repeated follow-up. Reasons for not seeking ART services included not feeling sick, need for multiple visits to the clinic, and long waiting times. 61.8% of the PWID underwent HB testing. Of the 2106 PWID eligible for HB vaccination, 81% initiated the vaccination schedule, but only 29% completed all three doses, despite intensive follow-up by outreach workers. PWID took an average of 8 clean needles-syringes/PWID/year over the project duration, with a mid-project high of 16 needles-syringes/PWID/year. PWID continued to also procure needles from other sources, such as chemists. One hundred five PWID were referred to OST services and 267 for rehabilitation services.

**Conclusions:**

A comprehensive HIV prevention, treatment, and care package is challenging to implement. Extensive efforts are needed to ensure the uptake of and retention in services for PWID; peer educators and outreach workers are required on a continuous basis. Services need to be tailored to client needs, considering clinic timing and distance from hotspots. Programs may consider provision of ART services at selected drop-in centers to increase uptake.

## Background

India has a large PWID (people who inject drugs) population estimated at 177,000 nationally with an estimated HIV prevalence of 7.2%. In Delhi, the HIV prevalence is estimated to be 18.1%, which is the second highest in the country [1, 2]. PWID continue to engage in unsafe injection and sexual behaviors resulting in new infections [3–5], highlighting the need for continued focus on HIV prevention while providing care services for HIV-positive PWID.

WHO, UNODC, and UNAIDS recommend a comprehensive package for the prevention, treatment, and care of HIV among PWID [6]. The package includes the following nine components: (i) needle-syringe program (NSP); (ii) opioid substitution therapy (OST) and other drug dependence treatment; (iii) HIV testing and counseling (HTC); (iv) antiretroviral therapy (ART); (v) prevention and treatment of sexually transmitted infections (STI), (vi) condom provision for PWID and their sex partners; (vii) targeted information, education, and communication for PWID and their sex partners; (viii) vaccination, diagnosis, and treatment of viral hepatitis; and (ix) prevention, diagnosis, and treatment of TB. To date, there is little documentation of the comprehensive package for prevention, treatment, and care of HIV, as well as the associated costs, based on real-world implementation, particularly in low resource settings. A search for published material on the topic in India only extracted one study carried out by UNODC that examined access to the nine components of the comprehensive package in north-east Indian states [7]. North-east states are ethnically and socio-culturally distinct, and the porous border with neighboring countries makes the drug availability and practices very different from the capital [8, 9].

In May 2011, the Population Council, in collaboration with the Sahara Center for Residential Care and Rehabilitation (Sahara), launched the AVHI (averting HIV infections) project for PWID in Delhi, as part of a HIV incidence study. A cohort of PWID provided the comprehensive HIV prevention and care services until April 2014. An integrated biological and behavioral assessment was conducted at three time points during this period to provide data on injection and sexual behaviors, HIV prevalence [8] and incidence [5], and the prevalence of hepatitis B and C [10] as elaborated in the “Methods” section. In this paper, we describe the uptake of services and related costs of implementing a comprehensive package for HIV prevention, treatment, and care services in a resource poor setting.

## Methods

From May to October 2011, PWID were recruited through targeted outreach, peer-referral, and as walk-in clients at five drop-in centers (DICs) operated by Sahara in central, east, north-east, and north-west districts of Delhi [11] for the longitudinal HIV incidence study designed to compare HIV incidence before and after the provision of comprehensive HIV prevention and care services. PWID who had injected atleast once in the last 3 months were eligible to participate. The study entailed three rounds of data collection: baseline at recruitment, follow-up visit 1 (FV1) approximately 1 year after the baseline (March–October 2013), and follow-up visit 2 (FV2) a year later at the end of the study (March–October 2014). At each visit, participants completed a behavioral survey and HIV testing (only HIV-negative PWID were tested at FV1 and FV2). At FV2, participants were asked open-ended questions regarding reasons for using/not using NSPs, or ART services. A variety of services were offered to participants during the course of the study. The period between baseline and FV1 served as the control phase when basic services were provided, and the period between FV1 and FV2 as the intervention phase when comprehensive services were provided to study participants.

HTC was offered to all PWID at baseline. From baseline to FV1, PWID received abscess care, outpatient medical consultations for common ailments, bathing/hygiene facilities, and nutrition services (tea/snack) at the DIC; they could access harm reduction and care services routinely available in the community throughout the project period. PWID who tested HIV positive at baseline received an accompanied referral to a government ART center for treatment. These services continued until the end of the project. Participants returning for their FV1 completed study procedures (testing for HIV, hepatitis B and C, and a behavioral survey; a subsample underwent STI testing) and started receiving the remainder of the comprehensive HIV prevention package (Information Education and Communication (IEC), NSP, condoms, hepatitis B vaccination for those found to be hepatitis B surface antigen (HBsAg) negative, STI treatment, OST through referral to government-approved centers, harm-reduction education, and referral for drug dependence treatment/rehabilitation). Participants could access these services at any of the five AVHI DICs. Distribution of needles/syringes and condoms, and abscess dressing was also offered through mobile vans parked at designated high-volume hotspots at specific times. The AVHI project provided these services until April 2014.

As part of the monitoring and evaluation, we collected daily data on various services provided to PWID enrolled in the study. Separate registers were maintained at each of the five DICs for each service provided, and detailed records were kept of all costs incurred. Program monitoring data and services statistics were entered into a central database. Cost data were based on monthly expenditures. Average prevalent exchange rate of USD 1 = INR 52 was used for the project. For the purpose of this analysis, cost data has been divided into: (i) capital costs which included procurement of furniture and medical equipment and other setup costs; (ii) recurring infrastructure and management cost that included rent and other utilities and personnel costs for the project manager, project accountant, site manager and security guards, and (iii) services provided. Personnel time for frontline services providers nurses, doctors, and outreach workers (ORWs) were divided per proportionate work allocation for each of the services. The methodology used for deciding the breakdown of costs into services components and other costs, including infrastructure and management, was based on the UNAIDS costing guidelines for HIV prevention strategies [12]. Capital costs were annualized using a discount rate of 3%. Capital goods were assumed to have a life of between 5 and 10 years depending on the item. Costs incurred for research activities and technical experts were excluded so that the costs mentioned in the paper reflect only the costs of provision of a comprehensive HIV prevention and care package.

## Results

A total of 3774 PWID (males: 3748 and females: 26) were enrolled into the study. Overall, 69% (*n* = 2604) of PWID were followed until the end of the study; 75.8% (males: 2480; females: 21) attended at least one follow-up visit while 59.4% (males: 2225; females: 17) attended both follow-up visits. Nine hundred eight male PWID did not attend any follow-up visits: 44 refused to participate, 337 moved out of Delhi, 49 were incarcerated, 10 were in long-term rehabilitation, 123 were confirmed dead, and 345 were untraced. Five female PWID did not attend any of the follow-up visits: 2 moved out of Delhi, and 3 died during the study period.

Table 1 details the socio-demographic profile of the participants at baseline. Nearly half (48.9%) of the participants were illiterate, most (51.7%) were never married, nearly half (46.6%) came from the three states around Delhi (Haryana, Rajasthan, and Uttar Pradesh), and 40.5% lived on the streets. The mean age at initiation of drug use (any drug) was 18.9 years (SD: 7.5 years); 78.1% had been using drugs for 6 years or more (data not shown). 92.7% had injected atleast once in the last 1 month. Among those who had injected atleast once in the last 1 month, the mean number of injection days was 19.8 (SD: 11.1).

**Table 1:** Baseline characteristics of participants

	Total *N* = 3774 %
Socio-demographic characteristics	
Education	
Illiterate	48.9
Class 1–6	25.5
Class 7 or higher	25.5
Marital status	
Married/cohabiting	36.7
Never married	51.7
Divorced/widowed	11.6
Religion	
Hindu	61.5
Non-Hindu	38.5
Regional origin	
Delhi	23.2
3 states adjacent to Delhi^a^	46.6
Other states	30.2
Accommodation	
Living in own or rented accommodation	59.5
Living on the street	40.5
Injecting behaviors	
Duration of injecting drug use	
1 year or less	34.3
2–5 years	40.9
6 or more years	24.8
Risky injection practices in the last 1 month	
Used needle/syringe used by others	
Never	71.9
Sometimes/most of the times	28.1
Injected drugs using front loading/back loading/splitting	
Never	80.9
Sometimes/most of the times	19.1
Shared other injecting equipment (vial/cooker/container, cotton/filter, or rinse water) with others	
Never	79.3
Sometimes/most of the times	20.7
Received pre-filled injection	
Never	87.3
Sometimes/most of the times	12.7
Drew drug from a common container	
Never	49.7
Sometimes/most of the times	50.3

^a^Haryana, Uttar Pradesh, Rajasthan

### Services

Table 2 summarizes program service components, uptake of services, and related costs. Research costs have been excluded from program costs.

**Table 2:** AVHI services, uptake, and cost of services

	Total cost (INR)	Number of PWID receiving service	Number of months service provided	Average cost per PWID per year (INR)	Description of program costs
Services under comprehensive package For prevention, treatment, and care Of HIV					
Needle and syringe program (NSP)	3,834,475	2508	26	705.65	Needles and syringes, alcohol swabs, waste disposal, ambulance service including driver and fuel costs (proportionate %), proportionate time for ORWs, and monitoring of the NSP.
OST referral	86,003	105	22	446.77	Nurses’ time for counseling
HIV testing and counseling^a^	4,455,294	7334	32	227.81	All HIV testing using rapid tests at baseline, and 4th generation AgAb tests at FV1 and FV2. HIV testing costs include laboratory cost for testing, phlebotomy costs, personnel costs (nurse) for post-test counseling, and proportionate share of waste disposal costs.
Referral for ART services and TB testing	2,611,195	1128	36	771.63	Nurse’s proportionate time for counseling and referral, proportionate % of ORW time, and travel costs for following up and escorting PWID.
STI check-up and treatment	1,395,235	792	36	587.22	Medications given to patients with symptoms and those who tested positive on laboratory test (including unused inventory), doctors’ and nurses’ time (proportionate %), monitoring services, ORW time, and transport costs (proportionate %) to bring back PWID for results and treatment.
Condom distribution	30,800	2392	26	5.94	Each participant accessing the NSP were offered two condoms at each visit.
Education and awareness activities	5,471,834	2840	26	889.25	Educational film, educational materials for ORWs, community events, street plays, ORW time, travel costs to visit PWID in the community, and cost for procuring educational films from other sources.
Hepatitis B testing	1,014,386	2331	12	435.17	Laboratory test Hepatitis B surface Antigen (HBs Ag), proportionate costs for phlebotomy and sample collection, proportionate time of nurses to provide pre- and post-test counseling, and waste disposal.
Hepatitis B vaccination	1,210,284	1706	26	327.43	Vaccination (vaccine, needle syringe, and alcohol swabs), proportionate time for nurses providing vaccination, proportionate ORW time, and travel costs for locating and reminding PWID to return for their second and third doses, and costs related to monitoring participant follow-up.
Hepatitis C testing	5,286,627	2330	12	2,268.94	Cost for Hepatitis C IgM/IgG Antibody tests and confirmatory RNA PCR tests. Counseling costs have been included in Hepatitis B counseling.
Abscess care	2,324,253	1219	36	635.56	Dressing materials, medications (antibiotics, non-steroid anti-inflammatory drugs, and pain killers), ambulance service including driver and fuel costs, proportionate time for medical officer, nurse, and ORW in the ambulance, and waste disposal.
Other Essential Services					
Nutrition	5,756,094	3494	36	549.14	Lunch packets, tea and biscuits, proportionate time for ORWs
Hygiene	722,688	2941	36	81.91	Bathing kits, proportionate time for ORWs
Medical consultation	2,994,704	1915	36	521.27	Medications and proportionate time for the doctor and nurse.
In-patient care and referral for detox/rehab	426,203	267	22	870.69	Cost of service provided by referral NGO per their rates

*ORW* outreach worker, *AgAb* antigen-antibody, *IgM/IgG* immunoglobulin M-immunoglobulin G, *RNA* ribonucleic acid, *PCR* polymerase chain reaction

^a^Indicates number of HIV tests, not number of PWID. PWID underwent HIV testing three times; the national program requires testing every 6 months

#### HIV testing and counseling

On recruitment, HIV testing was offered to all participants using HIV rapid tests per NACO guidelines [13]. Repeat HIV testing was conducted for all HIV-negative participants at FV1 and FV2, and positive test results were confirmed with Western Blot (WB) or PCR tests per the study protocol [5]; the associated costs for WB/PCR tests have not been included in program costs, as these were required to confirm new infections for the HIV incidence research component. A total of 3597 (95.3%) PWID were tested for HIV at baseline and received their test result and post-test counseling the same day [8]. One thousand nine hundred two HIV-negative PWID were tested at FV1 and 1842 HIV-negative PWID at FV2 (not all HIV-negative PWID returned for their FV1 and FV2). A total of 7334 HIV tests were undertaken over the study period at cost of INR 4,455,294. The average unit cost/PWID/year for HIV testing and counseling was INR 228 (USD 4.4).

#### Nutrition, hygiene, abscess care, and medical consultation

Supplementary nutrition, hygiene (bathing), abscess care, and medical consultation were provided all through the project period (36 months). Medical consultations included mainly common ailments such as diarrhea and respiratory and skin infections. In all, 3494 (92.5%) PWID used nutrition services and 2941 (77.9%) PWID used bathing services over the project period. The average unit cost/PWID/year was INR 82 (USD 1.6) for providing hygiene services and INR 549 (USD 10.5) for providing nutrition services.

A third of the PWID (32.3%; *n* = 1219) accessed abscess care services. However, of those, only 57% (*n* = 692) completed their abscess care with wound healing, while the remaining discontinued treatment midway. Overall, 62% (*n* = 760) of PWID received systemic antibiotics in addition to local wound care and the average number of dressing/treatment days per PWID were 8.7 days. Abscess care services cost the program INR 2,324,253, with an average unit cost/PWID/year of INR 636 (USD 12.2). Medical consultation services were used by 1915 (50.7%) PWID at a total cost of INR 2,994,704. The average unit cost/PWID/year was INR 521 (USD 10.0).

#### Hepatitis B testing and vaccination

The national HIV prevention program for PWID does not currently include HBV testing or vaccination. The AVHI project provided both these services. All participants returning for their FV1 visit were offered HBV testing using HBsAg tests, an indicator of active hepatitis B infection. Those testing HBsAg negative were offered three doses of hepatitis B vaccination at 0, 1, and 6 months. Although PWID were given appointment cards for their next dose, the vast majority misplaced their cards and needed repeated reminders. DIC nurses reviewed appointments every week and provided ORWs with lists of PWID due for their second or third vaccination doses. Active outreach and follow-up were undertaken over several months to encourage PWID to return to collect their HBV test result and to return for their second and third doses. Nurses also provided pre- and post-test counseling specific to hepatitis transmission, prevention, and care, including a referral to a government facility for management of active hepatitis B.

61.8% (2,331/3774) PWID underwent HBV testing, and 80% (*n* = 1875) returned to collect their HBV test results. Two thousand one hundred six (90.3%) PWID were negative and eligible for vaccination. Vaccination was initiated for 1706 (81%) PWID, and of those, only 492 (29%) completed all three doses. Program costs for undertaking HBV testing were INR 1,014,386 with an average unit cost/PWID/year of INR 435 (USD 8.40). The program cost of delivering vaccination services was INR 1,210,284 with an average unit cost/PWID/year of INR 327 (USD 6.3).

#### Hepatitis C testing, counseling, and referral

The national HIV prevention program for PWID does not currently include HCV testing or treatment. At FV1, the AVHI project offered HCV testing, prevention counseling, and referral to a public sector hospital for further management of active HCV infection. The HCV total antibody test was used to detect HCV antibodies followed by an RNA PCR test to confirm active infection. 61.7% (2,330/3774) PWID underwent HCV testing, 1655 (71%) PWID tested positive on the antibody test, and 1636 PWID underwent HCV PCR testing (19 did not have sufficient sample for testing).

53.5% (*n* = 1237) had active HCV infection that required medical management [10]. Nurses provided pre- and post-test counseling specific to hepatitis C transmission, prevention, and care. PWID with positive RNA PCR tests were referred to government hospitals for further management. The total program cost for undertaking HCV testing was INR 5,286,627 with an average unit cost/PWID/year of INR 101,666 (USD 43.6).

#### Needle and syringe and condom distribution program

The AVHI project provided sterile needles and syringes at DICs and in the community through a mobile van parked at designated hotspots at specific times on different days of the week. This service was provided to all participants after they completed their FV1 visit. Up to four needles and syringes were provided in exchange for used needles and syringes up to March 2013; thereafter, as clients found it difficult to carry used needles, the exchange component was discontinued and needles and syringes were distributed without requiring the return of used ones. Each participant accessing the NSP were offered two condoms at each visit.

A total of 2508 (87.7% of those who attended FV1) PWID accessed AVHI’s NSP, and received an average of 199 needle-syringes/PWID over 25 months of implementation (7.96 needles-syringes/person/month). The NSP program cost a total of INR 3,834,475 over the duration of the project and distributed a total of 501,391 needles-syringes. A total of INR 30,800 was spent for purchasing condoms, and 2392 PWID had taken condoms at least once. Costs related to condom distribution include only cost of condoms as these were distributed alongside needles-syringes.

**Fig. 1:**
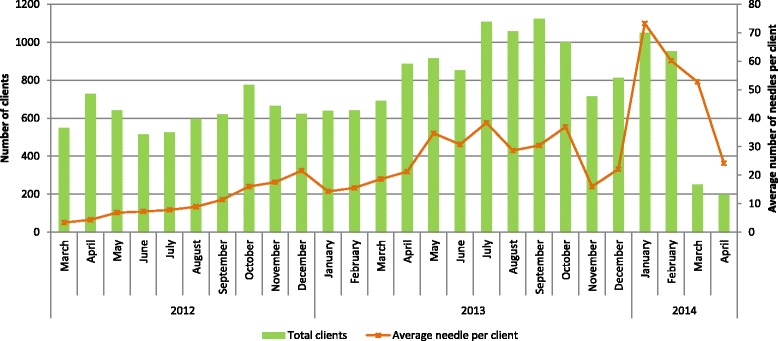
Monthly average of needle and syringe uptake through AVHI. Note: Project started by distributing two NS/person/day. In October 2012, number of NS given out increased to four NS/person/day. April 2013, exchange criteria removed. December 4, 2013, assembly elections held. Services ended at three DICs in March 2014 and two DICs in April 2014

Figure 1 illustrates the uptake of needles-syringes over the project period among participants who accessed NSP services. The uptake varied over time—it was lower on very cold or very hot summer days, and during national day celebrations or around elections when the police undertook cleanup drives in Delhi and police presence was increased to maintain law and order. The project started by giving out 2 needles-syringes/person/day with exchange of used needles-syringes. In October 2012, the number of needles-syringes given out was increased to 4 needles/person/day resulting in an increase in uptake. Around April 2013, when the exchange criterion was removed, there was significant increase in both the number of clients accessing services and number of needles taken going up to an average of 16 needles-syringes/PWID/month. The dip in needle-syringe uptake in November–December 2013 occurred around Delhi Assembly elections. This was followed by an upsurge in January 2014. The gradual decline after February 2014 occurred as DICs ended services at the end of the project—three DICs closed in March and the remaining two DICs in April 2014.

In the behavioral interview at FV2, only 49% of PWID reported using NSP in the last 3 months. Among them, 39% had last obtained sterile needles-syringes from a chemist/pharmacy, 38% from the AVHI project, 19% from TIs, and 4% from other sources. Of those who had not accessed NSP (51%) in the last 3 months, 76% reported obtaining new needles-syringes from medical shops. The most common reasons for not accessing NSP included not injecting (32%), distance from services (18%), and being in jail or out of Delhi (12%). Among PWID who were interviewed at baseline, FV1, and FV2 (*n* = 2242), the proportion who reported not having injected in the last 1 month increased from 6.4% at baseline to 26.3% at endline (*p* < 0.01).

#### Education and awareness activities

HIV prevention education and awareness activities were conducted after FV1 and included harm reduction and safe-sex counseling delivered by study nurses, one-to-one and group contact by peer educators and ORWs at hotspots, screening of educational films at the DIC, and street plays in the community. While each PWID received one-to-one counseling after his/her HIV and hepatitis tests, group and community education activities were initiated after the majority (>90%) of participants had completed their FV1 behavioral survey.

Peer educators and ORWs were provided printed cards with key information on HIV transmission and prevention, HBV and HCV infections, OST, partner testing, STIs, and ART, to ensure correct and uniform messaging. The program developed an educational film on key messages for screening at DICs. Educational films were also obtained from the Department of AIDS Control (DAC) and screened at the DICs. The AVHI project conducted 41 street plays at large hotspots and 21 community events around major festivals. A total of INR 5,471,834 was spent on education and awareness activities and reached 2840 PWID.

#### OST and rehabilitation services

The project provided OST services through referrals to government-approved centers for PWID who expressed a desire to use OST. A total of 105 PWID were provided accompanied referrals for OST at an average cost of INR 447 (USD 8.60)/PWID/year. Several PWID accessed OST services on their own—the proportion of PWID who reported having accessed OST services in the last month increased from 6.4% at baseline to 14.2% at endline. Data on retention in OST program was not accessible.

For drug dependence treatment and rehabilitation services, the AVHI project referred PWID to NGOs providing services free of cost where possible. Overall, 267 PWID were referred for these services. In instances, where PWID accessed these services themselves, the families paid out-of-pocket for the service (costs not included). The AVHI project paid for drug dependence treatment/rehabilitation services for 105 PWID, incurring an expenditure of INR 426,203 yielding an average unit cost of INR 871 (USD 16.70)/PWID/year.

#### STI services

The AVHI project provided syndromic management of STIs based on standard program guidelines. A sub-cohort of PWID were tested for STIs (syphilis, gonorrhea, chlamydia trachomatis, and HSV-2) at their FV1 (*n* = 879) and FV2 (*n* = 733) visits to determine prevalence and incidence of STIs. Participants testing positive for any of the above mentioned infections were provided counseling and treatment. The costs for STI testing were considered research costs and have not been included.

Forty-four PWID sought treatment for a genital infection and were treated using syndromic management guidelines. A total of 748 participants were diagnosed with an STI based on laboratory testing at FV1 or FV2; of those, 61% (*n* = 456) returned to collect their test results and 65 PWID were treated and 88% (*n* = 57) completed their treatment. Among those who tested positive for herpes simplex virus 2 (*n* = 405), none required treatment as they did not have active lesions; they did receive safe-sex counseling. ORWs made special efforts to advise PWID to collect their test results and to return for treatment. A total of INR 1,395,235 was spent on STI care and treatment, with an average cost of INR 587 (USD11.3)/PWID/year.

#### ART and TB services

All HIV-positive PWID were provided accompanied referrals to the nearest public health ART center where this treatment is provided free of charge. TB services have been categorized with ART services, as all HIV-positive PWID undergo TB testing as a part of their clinical assessment for ART initiation. During post-test counseling at the AVHI study visit, HIV-positive clients were given appointments at the DIC for ORWs to escort clients to the ART center. As PWID often missed these appointments, in the latter part of the project, ORWs contacted sero-positive clients in the community and directly escorted them to Integrated Counseling and Testing Centers (ICTCs) and ART centers—per NACO guidelines, it is mandatory to undergo HIV testing at a government ICTC to be eligible to access ARV services.

All participants (*n* = 1128) who tested HIV positive during the project were counseled for ART, and 84% (*n* = 945) of them received a referral. A total of 307 PWID were accompanied to ICTCs, and 201 PWID registered for ART services (17.8%). It took an average of 8 visits for HIV-positive PWID to complete registration procedures. Additionally, 46 HIV-negative PWID were referred for TB testing. At the end of the study, a total of 82 participants were receiving ART (endline survey). Based on focus group discussions with PWID, the main reasons for not accessing ART services included not feeling sick enough to start medications, repeated visits required to complete the registration process, long waiting time at ART centers, and not being able to reach the DIC on time for ORWs to escort them to ART centers. Those who registered successfully were appreciative of the assistance provided through accompanied referrals where ORWs helped them navigate the hospital system. The project spent a total of INR 2,611,195 on this service for providing referrals to 1128 HIV-positive PWID. The average unit cost/PWID/year for the service was INR 771 (USD 14.80).

### Proportionate costs incurred to provide services

The AVHI project spent a total of INR 55,516,754 (USD 1,067,629.88) over 36 months of project implementation. The costs attributed to *capital* costs include costs associated with the setup of DICs and training. *Recurrent* project management costs include personnel costs of project manager, site managers, accountant, and security guards, rent, and utilities. All salaries and costs were pegged to the prices listed by NACO for targeted intervention (TI) programs. *Recruitment* cost includes proportionate percentage time of the ORWs and the peer educators used for supporting cohort recruitment at the start of the project. The project spent INR 625,366 (USD 12,026; 1.7%) on capital costs (annualized), INR 14,807,187 (USD 284,753.60; 26.7%) for recurrent project management costs, and INR 2,145,963 (USD 41,268.52; 3.9%) on recruitment of the participants. Provision of comprehensive HIV prevention and care services accounted for the largest proportion of program costs (49.9%) and supplementary services for the remaining 17.8%.

Provision of the comprehensive HIV prevention and care package was INR 7,301.36 (USD 140.41)/PWID/year (excludes extra services: nutrition, hygiene, medical consultations which are not routinely provided by programs). These costs do not include the cost of services provided through government centers, such as ART provision at ART centers, TB testing, and treatment and OST. HCV testing was the most expensive component of the comprehensive package as it included HCV RNA PCR testing to identify active infections requiring medical management. Excluding HCV testing, the cost of provision of comprehensive services was INR 5,032.42 (USD 96.78)/PWID/year.

## Discussion

This paper presents service implementation data for a comprehensive package for the HIV prevention, treatment, and care services provided for PWID in Delhi. We provide a detailed component-wise breakup of costs and uptake of services in India; lessons learnt from this experience can inform other programs. The project was unique in that it was the first in the country to provide testing for hepatitis B (HBV) and hepatitis C (HCV) virus and hepatitis B vaccination for HBV negative PWID. Project Orchid [7] implemented in 13 districts in Manipur and Nagaland in north-east India and the CARE-SHAKTI HIV prevention program for PWID implemented in neighboring Bangladesh [14] are two programs that provided comprehensive harm-reduction services for PWID (NSE, abscess management, treatment of STIs, education, and condom distribution) and serve as comparators for our study. Both programs referred clients for treatment of HIV (ART), TB, and OST to government run programs. Neither of the two referenced programs provides testing for HBV/HCV nor HBV vaccination.

The needle-syringe distribution offers important lessons. The service was initiated with a needle exchange component and distribution of 2 needles-syringes/person/day from fixed facilities and mobile vans. This was rapidly changed to the distribution of 4 needles-syringes/day in response to client needs. The exchange component had to be removed as clients found it difficult to carry used needles, following which, a surge in uptake was observed. Despite all this, we were able to distribute an average of 96 needles-syringes/PWID/year which is lower than the 300 needles-syringes reported from Bangladesh [14] and 192 needles-syringes reported from Project Orchid [7] or the internationally recommended target of 140 needles-syringes [6]; both projects had exchange-based distribution. It is important to note that uptake increased slowly as more clients became eligible to receive needles-syringes after completing their FV1 reaching a high of 16 needles-syringes/PWID/year for a few months when all five DICs were at peak performance. The uptake of needles-syringes also had a seasonal variation and distribution through mobile vans served to enhance uptake. Our survey revealed that PWID in Delhi sourced needles from multiple sources, notably chemists (39%) from where they obtain pharmaceutical drugs, and other TI programs (19%), whichever was closer or convenient; it is well known that in Delhi chemists throw in free needles-syringes with the purchase of pharmaceutical drugs [15]. Taking into account the supply from chemists and other TI programs, it is probable that the actual use of clean needles-syringes among our clients would be higher. Our cost of USD 13.55/person/year for needle-syringe distribution was lower than international estimates of USD 23–71 from UNAIDS for all variety of distribution systems [16].

The uptake of ART by HIV-positive PWID was a challenge. In the national program, ART services are provided at designated public sector health centers/hospitals that require a HIV test result from government testing sites that are often located away from the ART center; thus PWID from our project had to undergo repeat HIV testing to be eligible and then visit the ART center at another location. Despite offering accompanied referrals with outreach workers, only a small proportion of positive PWID were registered for ART. PWID found it difficult to remember appointments, make multiple visits to the center, and wait in queues. While the uptake of HIV testing was very high (95%) as testing was conducted at the DIC itself, getting onto ART was suboptimal. This is in sharp contrast to the Orchid program report that shows 98% of their opioid injecting HIV-positive individuals were registered in ART services [7]. The socio-cultural context of rural north-east of the country is very different from the low-income, densely populated urban setting of Delhi. The national program may consider provision of HIV testing and ART services at selected TI DICs as a pilot project, this would help with improving uptake of HIV testing and ART services, and better treatment compliance. During our project period, OST services were only offered at approved TI centers and there was a limit to the number of people who could receive OST. Thus, many persons referred to OST remained on waiting lists. However, at endline, 14.2% of the PWID reported that they had accessed OST services. This is similar to the 13.7% coverage reported from the north-east [7].

There is ongoing debate on the introduction of hepatitis testing and provision of HBV vaccination for PWID in India. HBV and HCV testing was offered by our project. Uptake of HBV and HCV testing was high, and the majority came back to collect their results, but did so after several reminders. HBV vaccination was initiated for the majority; however, despite intense follow-up and several reminders, less than a third completed all three doses. Completion rates are also low in western countries; in a recent US study, 58% of PWID initiating HBV vaccination completed three doses [17]. HBV vaccination is now a part of the national immunization program and can be easily integrated into TI services; however, programs introducing HBV vaccination need to have effective retention strategies in place.

Additional services such as nutrition (snacks), hygiene (bathing facilities), recreational space, and medical consultations were provided to retain clients in services, based on Sahara’s past experience with service provision for PWID. Both the other programs, in north-east India and Bangladesh, also provided hygiene and recreational space as retention strategies. Learning from our experience, several TI programs in other parts of the capital have introduced similar services.

The AVHI project spent a total of USD 1,067,629.88 over 3 years (2011–2014). The largest component of the cost, over half of the budget, went towards delivering comprehensive HIV prevention and care services. An additional 17.8% went towards supplementary services such as hygiene, nutrition, medical consultations, and recreational space that we consider essential to the program, as a substantial proportion (40%) of our participants were street-based [8]. The CARE-SHAKTI and Orchid projects also provided bathing facilities and recreational space as retention strategies. Peer support and outreach is a critical component of all harm-reduction programs that supports almost all services (provision of needles-syringes, abscess care, crisis management, IEC, follow-up of clients, and accompanied referrals). A small proportion of this expense, which was spent during the recruitment phase, has been budgeted as cohort recruitment cost; the rest of it has been proportionately budgeted into each service component that was supported by it. The cost the AVHI program spent for cohort recruitment will not be necessary for the ongoing TI programs as they already serve registered clients. Providing comprehensive HIV prevention and care services, including hepatitis B vaccination, excluding hepatitis C testing, based on our project experience costs USD 96/person/year when ART, TB, and OST are provided through government program. This is marginally lower than the USD 110/person/year reported from Bangladesh (1999–2001) [14]. The more recent Project Orchid (2011–12) reported a cost of USD 154/person/year (USD 67 to reach clients and USD 87 for field monitoring) to provide harm-reduction services to PWID across 13 districts in Manipur and Nagaland in their scale-up phase [7].

There are some limitations. A mixed model of comprehensive HIV prevention, care, and treatment services were provided in this program—harm-reduction services were provided by the NGO while ART, TB, and OST services were accessed from the government health system. For all services where we referred clients, we did not have access to retention, adherence, or cost data; this data could have permitted a more detailed analysis of services and costs. The project undertook HBV and HCV testing but had to rely on the public health system for management of active infection.

## Conclusion

In conclusion, this paper documents the implementation of a comprehensive HIV prevention and care program in a large urban city and provides important insights into the challenges and the facilitators for the program. A comprehensive HIV prevention package is challenging to implement. Extensive efforts are needed to ensure the uptake of and retention in services for PWID; peer educators and outreach workers are required on a continuous basis. Services need to be tailored to client need, taking into account clinic timing and distance from hotspots. Programs may consider provision of ART services at selected drop-in centers to increase uptake. This program was the first to implement hepatitis B testing and vaccination services and provides feasibility information for the national program.

## Abbreviations

ART: Antiretroviral therapy
AVHI: Averting HIV infection
DIC: Drop-in center
HBV: Hepatitis B virus
HCV: Hepatitis C virus
HTC: HIV testing and counseling
IEC: Information Education and Communication
NACO: National AIDS Control Organization
NGO: Non-governmental organization
ORWs: Outreach workers
OST: Opioid substitution therapy
PWID: People who inject drugs
STI: Sexually transmitted infections
TB: Tuberculosis
UNODC: United Nations Office on Drugs and Crime
WHO: World Health Organization
